# The Spectral Extent of Phasic Suppression of Loudness and Distortion-Product Otoacoustic Emissions by Infrasound and Low-Frequency Tones

**DOI:** 10.1007/s10162-021-00830-2

**Published:** 2022-02-07

**Authors:** Carlos Jurado, Man Yui Pat Chow, Ka Man Lydia Leung, Marcelo Larrea, Juan Vizuete, Alain de Cheveigné, Torsten Marquardt

**Affiliations:** 1 UCL Ear Institute, London, WC1X8EE UK; 2 Escuela de Ingeniería en Sonido y Acústica, Universidad de Las Américas, Quito, EC170125 Ecuador; 3 Laboratoire Des Systemes Perceptifs, CNRS UMR 8248, Paris, France; 4 Departement d’Études Cognitives, École Normale Supérieure, PSL University, Paris, France

**Keywords:** human cochlea, cochlear mechanics, low-frequency hearing, infrasound, biasing

## Abstract

We investigated the effect of a biasing tone close to 5, 15, or 30 Hz on the response to higher-frequency probe tones, behaviorally, and by measuring distortion-product otoacoustic emissions (DPOAEs). The amplitude of the biasing tone was adjusted for criterion suppression of cubic DPOAE elicited by probe tones presented between 0.7 and 8 kHz, or criterion loudness suppression of a train of tone-pip probes in the range 0.125–8 kHz. For DPOAEs, the biasing-tone level for criterion suppression increased with probe-tone frequency by 8–9 dB/octave, consistent with an apex-to-base gradient of biasing-tone-induced basilar membrane displacement, as we verified by computational simulation. In contrast, the biasing-tone level for criterion loudness suppression increased with probe frequency by only 1–3 dB/octave, reminiscent of previously published data on low-side suppression of auditory nerve responses to characteristic frequency tones. These slopes were independent of biasing-tone frequency, but the biasing-tone sensation level required for criterion suppression was ~ 10 dB lower for the two infrasound biasing tones than for the 30-Hz biasing tone. On average, biasing-tone sensation levels as low as 5 dB were sufficient to modulate the perception of higher frequency sounds. Our results are relevant for recent debates on perceptual effects of environmental noise with very low-frequency content and might offer insight into the mechanism underlying low-side suppression.

## **INTRODUCTION**

It has long been known that low-frequency tones can mask high-frequency probe tones across a large spectral distance (Wegel and Lane 1924). Subsequent studies suggested that this effect originates in the cochlea—the so-called low-side suppression has been observed in basilar membrane (BM) vibration (Ruggero et al. 1992; Cooper 1996; Cooper and Rhode 1996; Geisler and Nutall 1997), otoacoustic emissions (Zwicker 1981; Scholz et al. 1999; Bian et al. 2002; Marquardt et al. 2007), cochlear hair-cell potentials (Patuzzi et al. 1984; Russell and Kossl 1992; Cheatham and Dallos 1997), and auditory nerve responses (Sellick et al. 1982; Temchin et al. 1997; Nam and Guinan 2016). The effect is not unexpected, supposing non-linear interaction between probe and suppressor, given that the travelling wave evoked by the suppressor traverses basal high-frequency regions characteristic of the probe on the BM. The travelling wave amplitude increases as it approaches the suppressor’s characteristic place, which explains why the suppressive effect increases with spectral proximity between probe stimulus and low-side suppressor. In this study, we attempted to quantify the slope of the suppressor excitation pattern for the human ear.

Of particular interest to us is the hypothesis that very low-frequency sounds, barely perceptible by themselves, might have perceptible modulation effects on higher frequencies. This possibility is of concern to researchers who look for reasons behind complaints about environmental noise with strong low-frequency content (e.g., Leventhall 2004; Møller and Pedersen 2004; Pedersen et al. 2008; Lichtenhan and Salt 2013; Alves et al. 2015; Yamada et al. 2016). The free-standing stereocilia of inner hair cells (IHCs) make them sensitive to BM velocity, whereas the stereocilia of outer hair cells (OHCs) are embedded in the overlying tectorial membrane and thus are sensitive to displacement. This suggests that IHC-dependent detectability of a suppressor tone might decrease faster as its frequency decreases than its OHC-mediated suppression effects on a probe (theoretically, by 6 dB/octave; see e.g., Salt and Hullar 2010). Furthermore, very low frequencies lack a distinct characteristic place on the BM and unlike higher frequencies, do not benefit from the frequency-selective gain boost at the peak of the travelling wave. Hence, we chose to study suppression thresholds, as well as the spectral extent of suppression, for tones below 40 Hz.

The physics of the cochlea is complex, but insight can be gained from considering limits and simplifications. In the static regime, an inward displacement of the footplate causes fluid to flow up scala vestibuli, through the helicotrema, and down scala tympani, causing the round window to bulge. Intra-cochlear pressure is uniform throughout and determined purely by the round window stiffness: there is no pressure difference across the BM to displace it. In the dynamic regime for frequencies below 40 Hz, fluid acceleration requires a pressure gradient. The intracochlear pressure amplitude is largest at the stapes footplate, intermediate at the helicotrema and almost zero at the very compliant round window. Consequently, the pressure *difference* across the BM is largest at the base and declines approximately monotonically to almost zero at the apex, where the pressures either side of the helicotrema are practically equal (i.e., the helicotrema is a pressure shunt). Below 40 Hz, the pressure is in-phase throughout the cochlea, resulting in an in-phase displacement of the compliant BM along its entire extent. If the compliance of the BM were uniform from base to apex, its displacement would be largest at the base. In reality, the BM compliance increases exponentially from base to apex. Therefore, the displacement amplitude caused by such low-frequency tone is larger at the low-frequency apex than at the high-frequency base. As a result, probe suppression effects produced by the suppressor-induced BM displacement are expected to decrease as the characteristic place of the probe shifts basally with increasing probe frequency. This has indeed been observed for distortion-product-otoacoustic emission (DPOAE) suppression (that reflects mechanical effects on the OHCs; see e.g., Marquardt et al. 2007) and for loudness suppression (that reflects effects on auditory nerve activity; see e.g., Zwicker 1977; Marquardt and Jurado 2018). Nonetheless, to our knowledge nobody yet has quantified the slope at which these two suppression effects decrease with increasing probe frequency. In the following, we explain our general approach of how we attempted this.

Suppressor tones that have periodicities far longer than those of the probe stimuli are often considered to provide a quasi-static “biasing” of the BM position and are therefore often called biasing tones (BTs). A general feature of this biasing is that, as the BT level is gradually increased, the effect is at first a phasic suppression of the high-frequency stimulus response, that then gives way to a tonic suppression at the highest suppressor levels. Phasic suppression can be observed at all stages of the auditory pathway, as long as the type of response can resolve the periodicity of the suppressor. It has been shown not only for brain responses (Gerull et al. 1991) but also perceptually: Zwicker (1977) demonstrated with his masking-period patterns that the masking of a short tone pip by tones < 40 Hz is dependent on the pip’s position within the masker cycle. Similarly, Marquardt and Jurado (2018) reported that very low frequency tones can periodically modulate the loudness of continuous tones.

A detailed physiological hypothesis for phasic suppression is commonly found in the literature on low-side suppression of DPOAE (Frank and Kössl 1996; 1997; Scholz et al. 1999; Bian et al. 2002; Lukashkin and Russel 2005; Drexl et al. 2012): the biasing of the BM position leads also to a bias in the normal resting angle of the OHC stereocilia, and consequently, in the resting current through their mechano-electrical transducer channels. This periodic displacement of the operating point along the mechano-electrical transducer’s sigmoidal input–output function is assumed to modulate not only the generation of intermodulation products (measurable as DPOAE), but also, as the operating point shifts into the saturating regions of shallower slope, it modulates the amplitude of the AC transduction current driving the OHC motility (for illustrations, see above literature). The associated periodic reduction in cochlear gain is likely to underly also the low-side suppression phenomena observed at the BM, IHCs and beyond, that show phasic suppression patterns similar to those of DPOAE suppression (Geisler and Nuttall 1997). The two methods described in the next section are based on this phasic suppression, and so we expected them to be equivalent in measuring indirectly the longitudinal gradient of the BM displacement.

Since the increase in suppression depth is nonlinearly related to the BM displacement, we take an iso-response approach: for each value of the probe frequency, the BT level is adjusted to reach a criterion suppression depth. We approximate the spatial pattern of BM excitation by repeating this adjustment for probe frequencies with characteristic places spanning the cochlea.

It should be noted that the convenient principle of scaling symmetry (Zweig 1976), often applied when estimating physiological responses from a single location, is not applicable for stimulation below 40 Hz, where the the pressure difference across the BM is largely shunted by the helicotrema and no travelling waves are generated. Here, with increasing frequency the BM displacement amplitude increases only because the pressure required to accelerate the fluid grows with 12 dB/octave, causing hearing sensitivity to increase more sharply in this range than for higher frequencies that elicit a travelling wave (Dallos 1970). An important advantage of keeping the suppressor frequency constant and changing the probe frequency is that we do not need to consider the 6-dB/octave difference between BM displacement and velocity when measuring the spatial pattern of suppressor excitation. Note, however, that we tested multiple suppressor frequencies (both above and below 20 Hz) to evaluate whether the slope with an infrasound suppressor is different to that of a suppressor in the audio frequency range, as this might be an explanation for numerous complaints about environmental infrasound.

## **MAIN EXPERIMENTS: LOW-SIDE SUPPRESSION OF DPOAE AND LOUDNESS**

Because the technique was already established in the Lab, we utilized DPOAE suppression, where we varied the frequency of the primary tone pair. It is a widely accepted assumption that the main generation site of (2f1-f2) DPOAEs is near the characteristic place of the f2 primary frequency (Brown and Kemp 1984; Martin et al. 1998). Thus, with increasing primary frequencies, the DPOAE generation site moves basally. Supposing that a fixed criterion of DPOAE suppression requires a fixed amount of BT-induced displacement, this should allow us to sample the longitudinal gradient of the BM displacement in response to the BT.

However, it became clear that the signal-to-noise ratio (SNR) of DPOAEs for f2 < 1 kHz was too low to quantify their suppression reliably, severely limiting the range over which we could sample the displacement gradient. For this reason, we decided to supplement this objective technique with a psychoacoustical procedure, during which participants had to adjust the BT level to achieve a constant phasic loudness suppression. Because we expected a priori that the suppression gradients of both DPOAE and loudness are based on the spatial BM displacement gradient, we hoped that the loudness-suppression method would allow us to extend the range of probe frequencies down to 63 Hz. Our results showed that this assumption was incorrect.

### Methods

#### DPOAE Suppression

The DPOAE suppression technique involves analyzing the suppression pattern of the cubic (2f1-f2) DPOAE, which is thought to reflect periodic changes in the operating-point position of the OHC’s mechano-electrical transducer channels, due to BM biasing (see, e.g., Scholz et al. 1999; Bian et al. 2002; Drexl et al. 2012). Marquardt and colleagues (2007) used this method previously to determine the frequency-dependence of low-frequency sound transfer from the ear canal to the BM, by adjusting the BT level so as to achieve a constant DPOAE suppression over a wide range of BT frequencies (15 − 480 Hz). In this study, we kept the BT frequency constant and varied instead the primary frequencies (0.5 − 8 kHz). This was done for 3 BT frequencies: 5, 15, and 30 Hz. Most other aspects of the DPOAE iso-suppression technique were as described in Marquardt et al. (2007).

The experiment was divided into two sessions on different days. The first session was used to individually optimize the primary tone parameters, so as to maximize the SNR. Various combinations of primary-tone frequency ratios (f2/f1 ~ 1.20 or 1.22) and level differences (L1‒L2 = 12, 15, or 18 dB) were systematically trialed for f2 tones in the range 0.5 − 8 kHz (preferably about half-octave spaced). The choice of primary frequencies was constrained to a (*n* × 5 + 2.5)-Hz grid (*n*: integer), in order to avoid spectral coincidence between the 2f1-f2 DPOAE (and its modulation side-lines) with harmonics of the BTs (which were multiples of 5 Hz). In order to keep the BM vibration in response to the f2 tones approximately frequency-independent, its sound pressure levels (SPL) were set according to the 50-phon curve (ISO 226, 2003), compensated by the free-field-to-eardrum pressure transfer function according to Shaw (1974). Each recording lasted 5.2 s. The SNR was considered sufficient when the DPOAE level was 25 dB over the local noise floor, which was determined by averaging over many of such spectra obtained during previous DPOAE experiments. Although sufficient DPOAE levels for the suppression measurements were found in almost all subjects, the f2 range where this SNR requirement was met was most often less than originally intended (0.5–8 kHz).

For each subject, the ear with the widest range was chosen for the subsequent DPOAE-suppression session. The continuous recordings were now increased to 20 s and sectioned into fifty 400-ms-long snippets. Artifact rejection excluded all snippets with powers that deviated from the median power by more than three scaled median-absolute deviations (Burke 1998). If more than 10 % of the snippets were rejected, the recording was repeated. To improve the SNR, the remaining snippets were averaged with the weighted method described by Hoke et al. (1984), where the weight of each snippet was its inversed power, and the weighted sum of all snippets was normalized by the sum of all weights. The spectrum of the averaged snippets as well as the average suppression pattern (i.e., the DPOAE amplitude as a function of BT phase; see details in Marquardt et al. 2007) were displayed immediately after each recording. Because the experimenter was inside the soundproof booth, the subject could also see the display, getting immediate feedback about noise (e.g., caused by moving, or loud breathing) and staying motivated by being able to follow the progress of the adjustment. The experimenter also monitored the probe microphone signal via headphones for noise artifacts. As subjects had no direct control over the suppressor level, it was furthermore important that the experimenter had direct verbal feedback as to whether the subject was still comfortable with it.

For each subject, the measurement order of the primary pairs was randomized. First, the unsuppressed 2f1-f2 level was reassessed and served as reference for the subsequent suppression adjustment procedure. In most cases, the repeatability was within 2 dB. Starting with the 30-Hz BT at a level below that expected to cause a large suppression, the BT level was iteratively adjusted by the experimenter until the difference between the un-suppressed reference DPOAE and the lowest DPOAE level within the suppression pattern was 6 dB. Typically, 3–4 attempts were required to obtain suppression patterns of slightly more and slightly less than 6-dB suppression (i.e., within 5 dB and 7 dB suppression), from which the BT level required for the 6-dB suppression was interpolated with an accuracy of 0.5 dB. After repeating this adjustment procedure also for the 15-Hz and 5-Hz BTs, the unsuppressed 2f1-f2 DPOAE level was reassessed. It was in 95 % of measurements within 1 dB of that at the start and always within 1.5 dB.

#### Phasic Loudness Suppression of Tone Pips

**Fig. 1:**
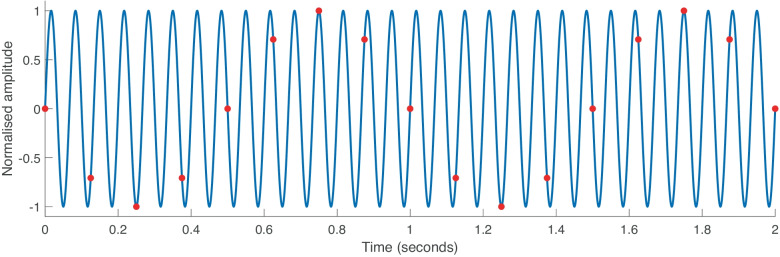
Continuous shift of the tone-pip probes (red dots) relative to the cycle of a 15 Hz BT. The probe repetition rate is 8 Hz. Two “beat” periods are shown

This suppression experiment is based on the masking-period pattern, a method devised by Zwicker (1977). He showed that the detection threshold of a short tone-pip strongly depended on its position within the cycle of a masking 20-Hz tone. Tone-pip thresholds were elevated if presented at moments of maximal BM displacement bias from its resting position but were little affected when presented while the BM was passing its resting position. In Zwicker’s experiments, the tone-pip repetition rate was equal to the biasing frequency. In contrast, we chose BT frequencies (*f*_*BT*_) that were 1 Hz away from multiples of our 8-Hz tone-pip repetition rate (i.e. *f*_*BT*_ = 8* N*-1; with *N* = 1, 2, and 4), so that the position of the tone-pip continuously shifted within the BT cycle, leading to slightly differing *f*_*BTs*_ than used for DPOAE suppression. For sufficiently high BT level, the loudness of the tone-pip train was periodically suppressed at a rate of 1 Hz, independent of the *f*_*BT*_ (see example in Fig. 1).

Using a jog wheel (PowerMate USB Multimedia Controller, Griffin Technology Inc., Nashville, TN), subjects were asked to adjust the BT level to a fixed suppression criterion to obtain iso-suppression curves for tone-pips with carrier frequencies in the range 63–4000 Hz (octave spaced). Each tone pip consisted of 3 cycles, including rise and fall cosine ramps of 1-cycle duration each. The jog wheel was configured so that a rotation of about 4° changed the sound level by 1 dB. For the safety of the participants, the maximum BT levels in these experiments were limited to 80 phon. A red light indicated to the subject when this limit was reached. The jog wheel’s push-button allowed the subject to control the progress of the procedure, so that the experimenter sat in these tests outside the booth.

Because the suppressibility of a probe tone by a BT decreases with probe-tone sensation level, all probe stimuli were presented at 15 dB SL. Therefore, individual tone-pip-train thresholds had to be measured in the first session. The subjects pressed the jog wheel to start the monaural presentation of a continuous, clearly audible tone-pip train. They then decreased the level by rotating the wheel anticlockwise, until the tone-pip train became just inaudible. During this initial phase, the subjects could turn the level repeatedly up and down to familiarize themselves with the jog wheel. They then pressed the jog wheel, to indicate that they were ready to start the procedure. This triggered a level decrease by a random step between 5 and 10 dB. Subjects now turned the wheel clockwise until the pip-train started to become audible again and then reversed direction. This change in direction made the level automatically decrease again by 5–10 dB, after which the subject had to turn the wheel clockwise until they started to perceive the pip-train again. This was repeated another 4 times. The median of the last four reversal levels, at which the subject triggered a downward random step, was taken as the detection threshold. As feedback on their performance, the standard deviation of these four reversal levels was displayed to the subjects. If it exceeded 2 dB, the measurement was discarded and repeated immediately. Otherwise, the procedure continued with the next probe frequency, which was chosen randomly. Once completed, the seven measurements were repeated three times, in new random orders. The median of the four measurements per probe frequency determined the final detection threshold. Note that due to the short duration of the tone pips, their detection thresholds were roughly 30 dB higher than those in ISO 389–2 (1994) so that the differences in peak levels between the 15 dB SL tone pips and the f2 DPOAE primary tone were on average only 8 dB.

During two further sessions, subjects adjusted the suppressor tone to a level so that the pip train became periodically suppressed. For this, they used here the same wheel-adjustment procedure they got already familiar with during the probe-threshold measurements. In case the pip train could not be suppressed at the 80-phon BT level limit, subjects double-pressed the jog wheel and the software skipped to the next condition. The suppression criterion was the perceived interruption of the pip train. Pilot tests had shown that this criterion was most robust: as the BT level was slowly increased, the complete suppression of a few tone-pips within the train turned the smoothly modulated pip train abruptly into a stimulus with a “galloping” rhythm. Before the procedure started, the experimenter demonstrated the criterion to the subjects using a mimicked interruption, by presenting pip trains that were sinusoidally-amplitude modulated at a rate of 1 Hz.

The lowest possible pip-train carrier frequency (*f*_*probe*_) for a given *f*_*BT*_ was limited by the duration of the 3-cycle tone pip relative to the BT cycle, so that *f*_*probe*_ > 8 × *f*_*BT*_. The suppression of probes was measured in ascending *f*_*probe*_ order for one *f*_*BT*_ at a time, where the *f*_*BT*_ order was randomized. Once loudness suppression levels were obtained for all conditions, the measurements were repeated three more times. For a given condition, the required suppressor level was obtained from the median of all four measurements.

#### Apparatus and Calibration

The experiments took place in a triple-walled soundproof booth at the UCL Ear Institute (ethics approval ID 0565/004), with identical experimental setup and calibration procedure for DPOAE and loudness suppression experiments. Signals were generated, recorded, and analyzed using MATLAB. Both experiments utilized a ER10C OAE probe (Etymotics Research Inc., Elk Grove Village, IL). Only one of its receivers generated the probe stimuli for the loudness experiment. The ER10C microphone served both in the DPOAE recordings and in-situ calibrations. The high-pass cutoff frequency of its microphone amplifier was increased to 1 kHz, in order to avoid overloading the A/D converter of the 24-bit multi-channel audio device (RME Fireface UC, RME Audio AG, Haimhausen, Germany) by the intense BTs. The latter were produced by a DT-48 earphone (Beyerdynamic GmbH & Co. KG, Heilbronn, Germany), whose acoustic output was delivered into the ear canal through a narrow tightly sealed polyethylene tube (200 mm in length, 0.5 mm of inner diameter) that pierced the ER10C-14A foam eartip. The thin delivery tube constitutes an acoustic low-pass filter, which together with a maximum voltage output limited by an RC low-pass filter and an attenuator (placed between the audio device and power amplifier), prevented accidental sound delivery above ~ 105 phon (extrapolated from ISO 226–2003, and below 20 Hz from Møller and Pedersen 2004). Before the experiments commenced, the transfer function of the ER10C microphone was measured in an artificial ear (Type 4157, Brüel & Kjær Sound & Vibration Measurement A/S, Denmark). This was used for the in situ calibrations and to correct the complex-valued spectra of the DPOAE recordings. The electrical signals were adjusted to achieve the desired sound pressures at the probe’s microphone.

#### Subjects

Twenty subjects (mean age = 29 years, 12 females) were recruited for the DPOAE experiment and nine subjects (mean age = 25 years, seven females) for the loudness-suppression experiment, seven of which also participated in the DPOAE experiment. No subject reported tinnitus, hypersensitivity to very low-frequency sounds, or a history of other hearing disorders. The absence of ear obstructions or excessive earwax were checked by otoscopy. Subsequently, normal middle-ear function was assessed by tympanometry.

Normal auditory function in the relevant frequency range was evidenced by either DPOAE measurements, or measurements of the probe’s detection threshold during the respective experiments. Two recruits from the DPOAE group were excluded, one lacking sufficient DPOAE levels (i.e., > 25 dB SNR) within an f2-range of at least two octaves, the other because their DPOAEs could not be suppressed. In the loudness-suppression group, across all probe frequencies no individual had a pip-train threshold of more than 12 dB above the group’s average and no one was excluded.

Detection thresholds for the three BTs (2000-ms duration, including 3-cycle on- and offset ramps) were measured every day of testing for each subject to check for normal sensitivity to these very low-frequency tones using a 3-down 1-up two-alternative-forced choice (2-AFC) adaptive procedure. None of the individuals presented thresholds exceeding 15 dB HL (*f*_*BT*_ = 30 Hz), or 9 dB above the reference thresholds proposed by Møller and Pedersen (2004) for the two infrasound tones. (A 3-dB correction accounted for monaural listening.)

#### Derivation of the Cumulative Average-Slope Curves

At the high-frequency end of the measurements, the BT often reached the maximum permissible levels so that data are missing, in particular for subjects that required generally higher suppressor levels. When simply taking the average of available individual data, this ceiling effect would have artificially flattened the group’s average curves at the high-frequency end. Because we still wanted to include the available high-frequency data, we derived the shape of the curve that combines the curves from all subjects via their local slopes.

For the loudness-suppression experiments, where the probe-frequencies lied on a fixed grid, averaging the individual’s local slopes was straightforward. The shape of the combined curve for the group (here called “cumulative average-slope curve”) was simply obtained by cumulatively joining the local sections of the averaged local slopes end-to-end.

However, due to the non-fixed grid of the f2-frequencies, the derivation of local slopes for the DPOAE iso-suppression curves was somewhat more complex: (1) The local slopes of every individual curve were calculated between all adjacent probe frequencies; the geometric mean frequency (of these adjacent points) was the position of the local slope values on the abscissa of the resulting local-slope scatter plot. (2) The cloud of slope *vs* frequency data from all subjects was then fitted with a locally-estimated-scatterplot smoothing (LOESS) curve (Cleveland et al. 1992). This gave a continuous curve of the local slope estimate with standard-error of the mean (SEM) estimates. (3) This curve was then resampled to 200 frequency points per octave and, similarly to the local loudness slopes, integrated in order to obtain the cumulative average-slope curve. A figure with such curves is shown in the discussion.

### Results

**Fig. 2:**
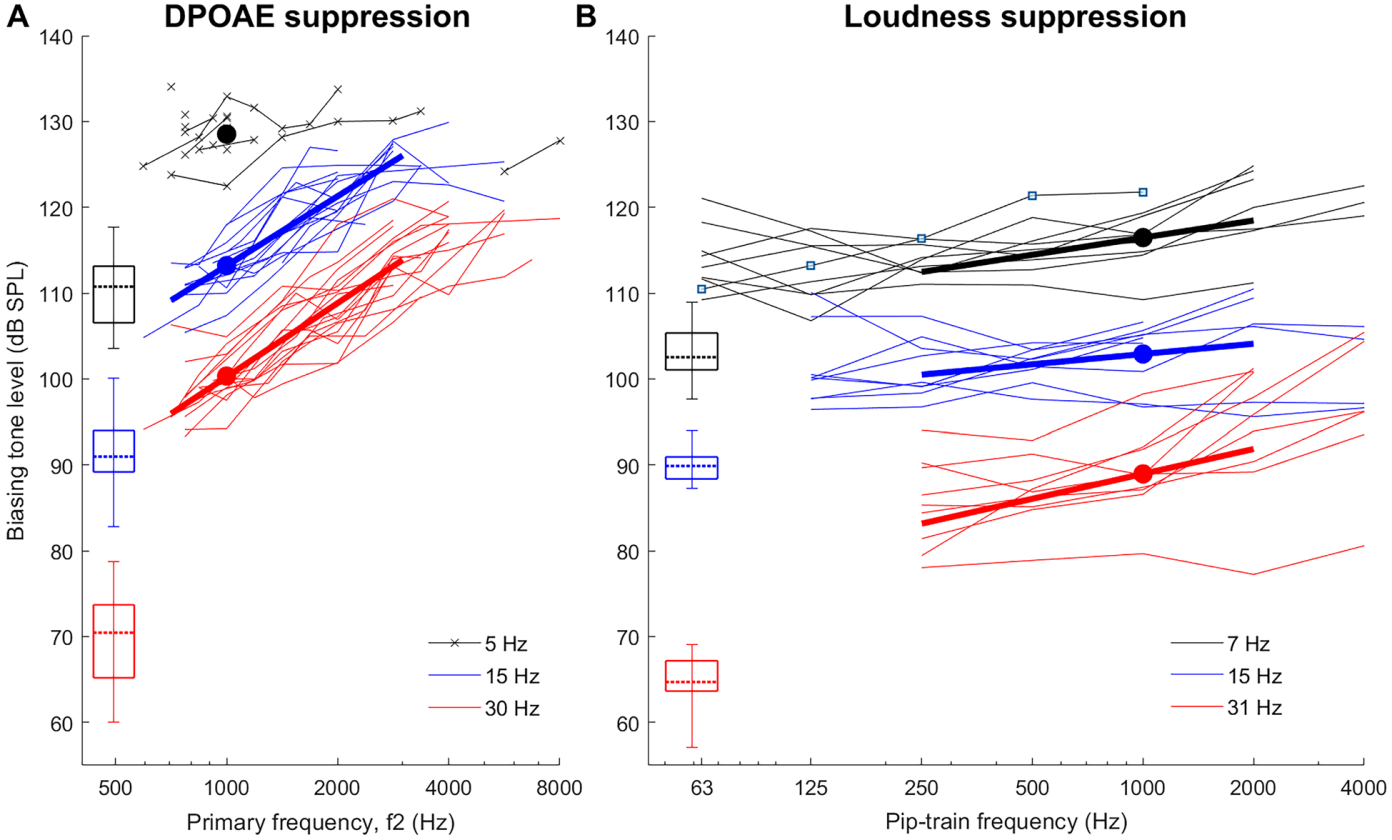
**A** Individual DPOAE suppression thresholds (biasing-tone level required to suppress the 2f1-f2 DPOAE by 6 dB) for biasing tones of 5, 15, and 30 Hz are shown for 18 participants. Bold lines are linear fits in the frequency range 0.7 − 3 kHz, where data were available from most subjects. The levels of f2 were set to 50 phon, and the f1 parameters were optimized for each f2 to maximize the 2f1-f2 DPOAE level. **B** Individual loudness suppression thresholds as function of pip-train frequency for the biasing tones of 7, 15, and 31 Hz. Bold lines are linear fits in the frequency range 0.25 − 2 kHz, where data were available for almost all subjects. Both panels: whisker plots show relevant statistics for the BT detection thresholds for the two subject groups participating in each experiment (minimum, maximum, quartiles, and median). Filled circles show average BT levels at 1000 Hz. For individual slope values, see Table 1

Figure 2 shows an overview of all individual DPOAE (panel A) and loudness iso-suppression (panel B) curves, with identical axes scales (dB/octave). As expected, to maintain a constant suppression with increasing probe frequency, an increase in BT level was required. However, the loudness iso-suppression curves are far shallower than the DPOAE iso-suppression curves. This finding was rather unexpected and led us to conduct the numerical simulations and control experiments reported in the subsequent sections.

As the 5-Hz BT required generally higher sound pressure levels that almost always reached the safety limit for f2 > 1 kHz, very few DPOAE suppression data could be obtained with this BT. Oddly, in one subject that had sufficient DPOAE levels with f2 = 0.9–8 kHz, the 5-Hz BT was able to suppress DPOAEs produced by f2 ≥ 5655 Hz, while not below this. With the BT frequencies of 15 Hz and 30 Hz, however, the pattern measured in this ear was like that of all other ears: BT levels generally required a progressive increase with increasing f2.

Safety limits were also reached for many subjects when attempting to suppress the highest frequency probes with the 15-Hz and 30/31-Hz BTs. Due to low SNR, very few DPOAE suppression thresholds were obtained with f2 < 0.7 kHz. This restricted the quantitative slope analysis for the individual DPOAE iso-suppression curves to a range 0.7–3 kHz, and for the loudness iso-suppression curves to 0.25–2 kHz. Because of the limited range available for the 5-Hz BT, slopes of these DPOAE iso-suppression data were not analyzed. Table 1 lists the slopes derived from linear fits through the data. All fits were based on probe-frequency ranges of at least two octaves (see individual curves in Fig. 2). The average slopes for the 15-Hz and 30-Hz DPOAE iso-suppression curves were 8.1 and 8.6 dB/octave, respectively. They were statistically indistinguishable according to a paired *t*-test (*T*(17) = 1.64, *p* = 0.12), and there was a notable correlation between individual iso-suppression slopes (*R*^2^: 0.66, *p* < 0.0001). In contrast, average slopes for the loudness-suppression curves were only 2.0, 1.2, and 2.9 dB/octave with the 7-, 15-, and 31-Hz BTs, respectively. For the seven common subjects to both DPOAE and loudness experiments, iso-suppression slopes for the 15-Hz and 30/31-Hz BTs were significantly different, according to a paired *t*-test (15 Hz: *T*(6) = 8.49, *p* < 0.001; 30/31 Hz: *T*(6) = 4.60, *p* < 0.01). As observed for the DPOAE data, the 15- and 31-Hz loudness-suppression slopes were again significantly correlated (*R*^2^ = 0.74, *p* < 0.01). The slopes for the 7-Hz BT were not significantly correlated with those for the two other BTs (7 vs. 15 Hz: *R*^2^ = 0.06, *p* = 0.52; 7 vs. 31 Hz: *R*^2^ = 0.17, *p* = 0.27). Despite the correlation between the slopes for the 15-Hz and 30/31-Hz BTs within, there was no significant correlation between the slopes across the two types of suppression, which indicates that different mechanisms might underly the two types of suppression (tested for the seven common subjects; 15 Hz: *R*^2^ = 0.13, *p* = 0.42; 30/31 Hz: *R*^2^ = 0.04, *p* = 0.69; see Table 1).

**Table 1:** Individual DPOAE suppression slopes and loudness-suppression slopes, determined by a linear fit in the frequency ranges 0.7–3 kHz and 0.25–2 kHz, respectively

	DPOAE-suppression slopes (dB/octave)		Loudness-suppression slopes (dB/octave)		
Subject	15-HzBT	30-HzBT	7-HzBT	15-Hz BT	31-Hz BT
1	10.2	11.3	3.6	0.7	3.7
2	9.6	9.2	2.7^a^	4.0	7.3
3	-	-	4.0	0.9	1.7
4	5.6	4.6	2.5	− 0.3^a^	2.3
5	8.5	8.4	0.4	0.2	− 0.4
6	7.8	8.5	0.9^a^	3.4	4.8
7	-	-	1.4	0.5	3.2
8	7.2	7.9	2.5	2.8^a^	3.8
9	7.1	8.9	0.1	− 1.3	− 0.3
10	11.3	11.0			
11	8.4	9.5			
12	11.5	12.8			
13	6.8	11.3			
14	8.5	7.6			
15	8.0	9.1			
16	8.1	7.6			
17	4.3	3.4			
18	7.3	9.3			
19	6.3	4.7			
20	8.8	10.3			
Mean (SD)	8.1 (1.8)	8.6 (2.5)	2.0 (1.4)	1.2 (1.8)	2.9 (2.4)

^a^Obtained only between 0.25 and 1 kHz, due to unavailable 2-kHzdata

Since the human ear is rather insensitive to very low-frequency sounds, it was of interest to relate the absolute sound pressure level of the BTs to sensation levels. Loudness suppression was generally achieved with much lower BT levels than DPOAE suppression, when expressed relative to the individual BT thresholds. The BT levels required to suppress the DPOAE for f2 = 1000 Hz were on average 18 (rough approximation, affected by ceiling), 21.4 and 30.1 dB SL for the 5-, 15-, and 30-Hz BTs, respectively. Average levels for loudness suppression of 1000-Hz tone-pip probes were 13.4, 12.9, and 24.1 dB SL for the 7-, 15-, and 31-Hz BTs, respectively. For the seven subjects who participated in both experiments, these sensation levels were significantly lower than those for DPOAE suppression (paired *t*-test for 15 Hz: *T*(6) =  − 5.45, *p* < 0.01; for 30 and 31 Hz: *T*(6) =  − 4.66, *p* < 0.01). A lack of significant correlation between their loudness- and DPOAE-suppression thresholds for 1000-Hz probes also indicates that different mechanisms might underly the two types of suppression (15 Hz: *R*^2^ = 0.06, *p* = 0.59; 30 and 31 Hz: *R*^2^ = 0.18, *p* = 0.35).

## **FINITE ELEMENT SIMULATIONS USING A COCHLEAR-BOX MODEL**

To investigate whether either of the two contradicting slopes has a connection to the longitudinal gradient of BM displacement, we decided to simulate the effects of our experiments with a finite element model of the cochlea. This model had already been implemented in COMSOL Multiphysics (COMSOL AB, Stockholm, Sweden) to study the effect of the helicotrema on cochlear acoustics.

Allen and Sen (2000) suggested a spatial gradient of 9 dB/octave for the basal-tail region of the travelling wave (~ 5 dB/mm for the human cochlea). This value is in close agreement with our DPOAE iso-suppression curves. Nonetheless, we did not know how the pressure field in the travelling-wave tail region relates to the pressure field generated by tones below 40 Hz, which do not evoke a travelling wave (see “Introduction”).

### Methods in Computer Simulations

**Fig. 3:**
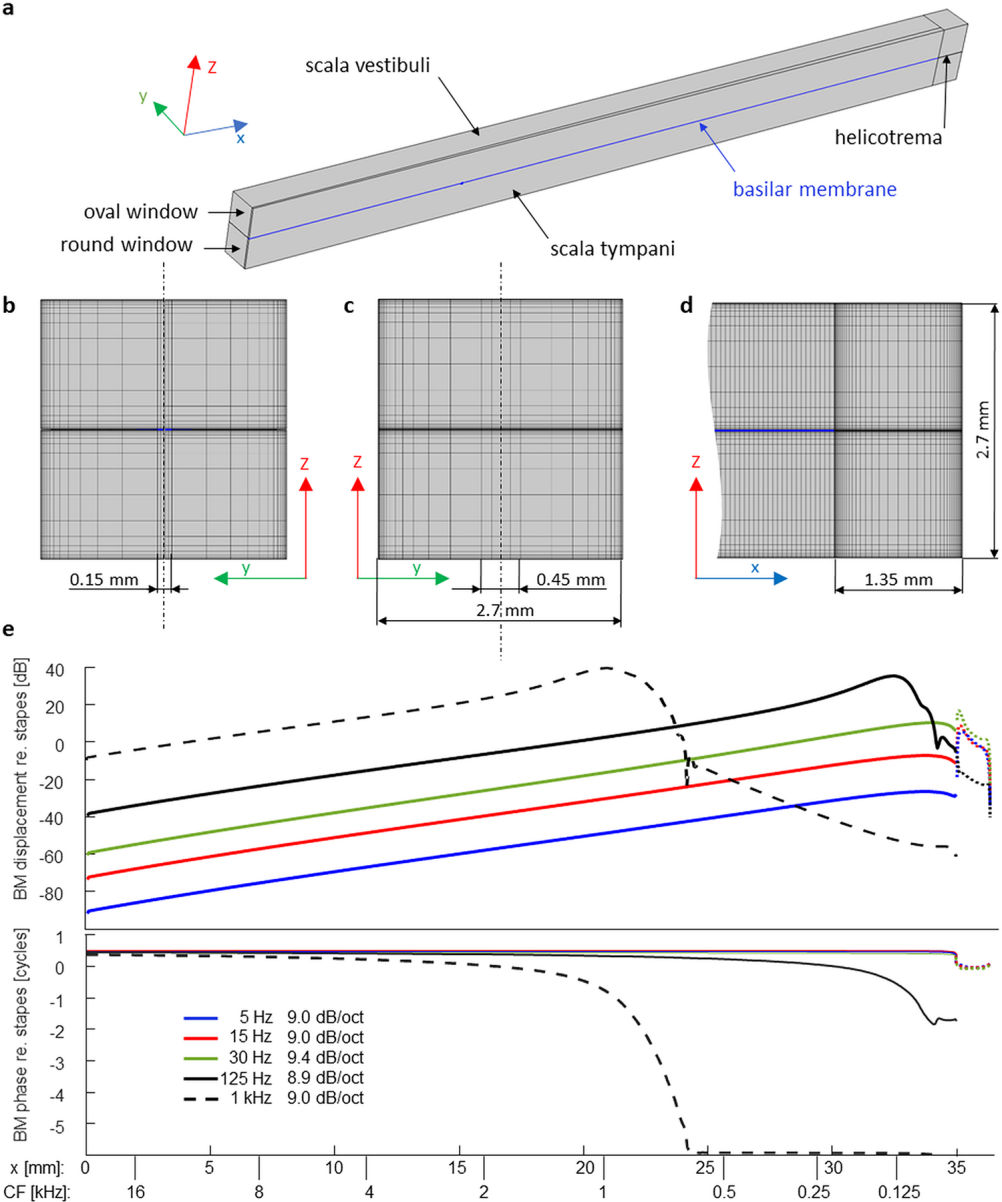
Finite element model of the human cochlea. **a** Fluid compartments (gray) and BM (blue) of half of the cochlear box model (i.e., half its width). Because its cross sections were symmetrical, only one half of the model had to be numerically solved. **b** View of the cross-sectional mesh in the *x*-direction. **c** View of the cross-sectional mesh in the opposite direction, showing the surface of the helicotrema compartment. The vertical dash-dotted lines in **b** and **c** indicate the model’s symmetry. **d** Lateral view of the mesh at the apical end of the (half) model shown in **a** in the *y*-direction, including the 1.35-mm-long helicotrema compartments. **e** Magnitude and phase of BM displacement along the cochlea in response to tones of various frequencies. The dotted lines beyond 35 mm represent fluid displacement inside the helicotrema. The legend in the lower panel gives also the slopes of the displacement expressed in dB/octave between the characteristic frequencies (lower abscissa scale) of 0.5 and 16 kHz (only 2–16 kHz for the 1-kHz tone), derived from the characteristic places for tonal stimulations. As can be seen in the lower panel, the BM moves in-phase along its entire length when stimulated at and below 30 Hz

Utilizing COMSOL’s Thermoacoustics Interface, the cochlea was implemented as an uncoiled box with two fluid compartments separated by a 35-mm long solid partition that represented the BM (Fig. 3a). The BM had the density of water, a width of 150 µm at base, 450 µm at the apex and a constant thickness of 10 µm. It was an orthotropic solid with a Young’s modulus in the longitudinal direction (*E*_*x*_) a thousand times smaller than across (*E*_*y*_). Along the longitudinal location (*x*), *E*_*y*_ was adjusted to give the model roughly the tonotopy of the human cochlea (Greenwood 1990), as can be seen by the dual scale of the abscissa in Fig. 3e. It was achieved by adjusting empirically the parameter values in the following formula that gives *E*_*y*_ in Pascals (The distance from the base (*x*) is given in meter.):

1 $${E}_{y}=5\cdot {10}^{7}{\left(1-\frac{x}{0.045}\right)}^{4.5}$$

The shear moduli were G_x_ = (E_y_/Pa)/50 N/m^2^, G_y_ = (E_y_/Pa)/10 N/m^2^ and G_z_ = 1 N/m^2^. The corresponding Poisson ratios were {0.005, 0.3, 0.005}.

A 1.35-mm-long compartment with a 0.45-mm-wide helicotrema was added to the apical end.

The mesh sectioned the BM longitudinally in 700 elements. The cross-sectional mesh of BM and fluid compartments was non-uniform to resolve the shear movement within the boundary layers more finely (Fig. 3 b and c). The meshing detail at the apical helicotrema is shown in Fig. 3d. Since the model was symmetric (with the BM and helicotrema symmetrically divided along their midline), all middle surfaces were given a symmetry constraint so that only one-half of the model needed to be solved (450,800 elements, 12,681,661 degrees of freedom).

The acoustic input was applied by defining a perpendicular harmonic displacement to the basal fluid surface labelled *oval window* in Fig. 3a. For very low-frequency tones, this displacement is proportional to the ear-canal pressure in the real ear (due to the stiffness-dominated impedance of the middle ear). The basal fluid surface labelled *round window* was unconstrained. All BM-fluid boundaries were given a non-slip fluid–structure interaction. The outer boundaries of the fluid had also a non-slip constraint. All losses were within the viscous fluid (i.e., the BM was not damped). The fluid had the mechanical properties of water.

The direct stationary solver PARDISO ran on a Dell PowerEdge R910 server (four Xeon CPUs E7- 4870 at 2.40 GHz, 40 cores total). The computation took approximately 3 h per frequency, requiring almost all of the available 1 TB RAM.

### Results

As expected, suppressor tones used in our experiments (< 40 Hz) produce no travelling waves and the model’s BM movement is in-phase along its entire length (Fig. 3e). The longitudinal gradient of the BM displacement is fairly independent of suppressor tone frequency and has a value of ~ 9 dB/octave. We included a couple of higher-frequency tones to show how the model simulates their travelling waves. Indeed, the BM-displacement gradient is in the wave-tail region 9-dB/octave as was previously predicted analytically by Allen and Sen (2000).

In conclusion, the simulations confirm that the BM displacement decreases at approximately 9 dB/octave from apex to base, which is consistent with our iso-suppression curve based on DPOAE suppression. This finding supports current theories about low-side suppression and confirms the assumptions on which our main experiments were based. We were, however, still left with the question why the loudness-suppression slope differs from the longitudinal gradient of the BM displacement.

## **CONTROL EXPERIMENTS: MODULATION DETECTION THRESHOLDS AND CONTINUOUS PROBE TONE**

Two possible issues were identified that might have affected our main perceptual measurements: (1) the criterion of full suppression was too extreme; (2) the probe duration of only 3 cycles was too short to fully engage the active cochlear processes which we thought that the suppressor tone would impede. Hence, two control experiments were conducted, focusing on the 15-Hz BT only. In the first control experiment, the response criterion was changed to a just-detectable 1-Hz modulation in the pip-train. As this criterion change did not appear to affect the slope, a second control experiment with continuous probe tones was run.

### Methods

The experiments took place in an audiometric cabin at the Acoustics Laboratory of Universidad de Las Américas (ethics approval ID 2020–0626). The setup used for these control experiments was very similar to the loudness suppression experiments described above, except that the BTs were generated using a DA270-8 10-inch aluminum-cone subwoofer (Dayton Audio, USA), with its front tightly sealed to an acrylic cover and connected to the ER10C-14A foam eartip via a longer silicon tube (0.8 m, ~ 0.7 mm inner diameter). Further, an RME Fireface-802 audio interface was used. The headphone output of the latter directly drove the BT source.

Twelve normal-hearing subjects were recruited (mean age = 23 years, 2 females). They reported no tinnitus, hypersensitivity to very low-frequency sounds, nor history of other hearing disorders. A total of four experimental sessions were carried out, each starting with 15-Hz detection-threshold measurements using the 3-down 1-up 2-AFC adaptive procedure as in the main experiment. No subject had a BT threshold exceeding 11 dB of those for 15-Hz tones proposed by Møller and Pedersen (2004; corrected for monaural listening). Tympanometry was not available. In addition, detection thresholds for both probe types (pip-train and continuous), ranging now 125 to 8000 Hz (octave spaced), were measured at the beginning of the first session twice. If a repeated measurement differed by more than 3 dB, a third measurement was performed. The average of the 2 or 3 measurements defined the probe’s detection threshold that allowed to set the levels of the probe stimuli to 15 dB SL. Interval durations were 1000 ms for the tone-pip trains and 600 ms for the continuous probe tones (the latter included two 4-cycle long cosine ramps). No subjects had continuous tone thresholds of more than 17 dB above ISO 389–2 (1994). Because the previously devised wheel-adjustment method required a considerable amount of training, modulation thresholds were now measured with a one-interval Yes/No paradigm, combined with a 1-up 1-down adaptive rule. Two adaptive tracks were run simultaneously, with the stimulus presentations for each track presented in random alternation. The stimulus interval contained both the BT and probe stimulus. Interval duration was 4000 ms for the tone-pip trains (1-Hz modulation frequency) and 1000 ms for the continuous probe tones (15-Hz modulation frequency). Subjects had to decide whether they heard the probe as modulated or not. The BT level was adapted according to their responses. The step size started with 8 dB and was reduced to 4 dB after two reversals. After two further reversals, the track continued for six further reversals with 2-dB steps. The average of these six reversal levels determined the track’s threshold. One run stopped after both tracks ended, and the run’s threshold corresponded to the average of both tracks. Two runs were performed, and if their thresholds differed by more than 3 dB, a third run was completed. Modulation threshold was obtained from averaging the outcome of these 2 or 3 runs. Before data collection, subjects underwent a practice period. All probe frequencies were measured in random order.

### Results

**Fig. 4:**
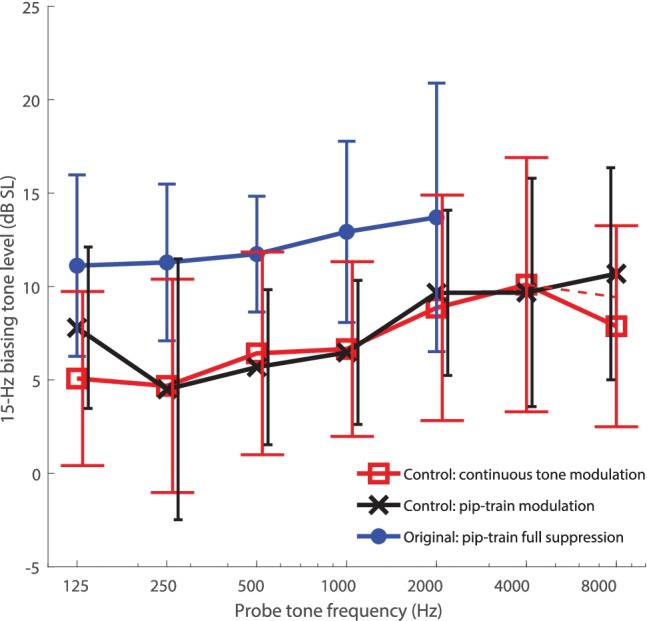
Modulation detection thresholds obtained in control experiments for pip-train probes (black) and continuous-tone probes (red) using a 15-Hz biasing tone. Data were averaged across the twelve new subjects. Also, data from the main experiment, obtained using a full-suppression criterion with a 15-Hz biasing tone, are re-plotted (blue). The criterion in both control experiments was the detection of a modulation. The error bars indicate ± 1 SD of the individual modulation thresholds (in dB SL) at each probe frequency. Note that the steep drop at 8 kHz in the continuous-tone curve is likely due to missing 8-kHz data from two subjects (for whom the safety limit was reached), as suggested by the local slope between 4 and 8 kHz for the remaining 10 subjects (red dashed line)

Figure 4 shows the group’s average modulation-detection thresholds (given in dB SL), obtained using the pip-train and continuous-tone probes. Average 15-Hz full-suppression thresholds from the main experiments are re-plotted here for easier comparison. As expected, the thresholds for modulation detection lay clearly below those for full suppression. Comparing among pip-train data only, the BT levels between 0.25 and 2 kHz were on average 6.1 dB lower, a highly significant difference (one-way ANOVA: *F*(1,82) = 30.4, *p* < 0.0001). Thanks to this, probe-tone frequencies up to 8 kHz could be tested for almost all subjects (pip-train: 11/12 subjects; continuous tone: 10/12 subjects) and the loudness-modulation data covered the full frequency range of the DPOAE suppression data.

The modulation-detection threshold levels obtained in the control experiments were generally similar for the two probe types, and the steepness of the resulting curves was again far lower than that of the DPOAE iso-suppression curves. The weaker dependence on probe frequency was similar to that observed using the full-suppression criterion in the original experiment. A two-way ANOVA, considering data from both control-experiments in the range 0.25–2 kHz, showed that the probe frequency was still a significant factor, while stimulus type was not [respectively: *F*(3, 88) = 3.31, *p* < 0.05; *F*(1,88) = 0.005, *p* = 0.94]. The average slopes of 1.7 and 1.4 dB/octave in this region, obtained respectively with the pip-train and continuous probes, were very similar to the 1.2 dB/octave observed with the full-suppression criterium in the main tests.

In summary, the control experiments confirmed the shallow slope observed in the original loudness-suppression experiment. The frequency range of these additional measurements also overlaps well with the frequency range of the DPOAE iso-suppression curves, strengthening the notion that there is a qualitative difference between low-side suppression of DPOAE and loudness.

## **GENERAL DISCUSSION**

Utilizing two non-invasive methods, we measured the spectral extent of low-side suppression in the human ear. Our initial assumption was that the two methods probed the same phenomenon, a shift of the operating point of the mechano-electrical transducer of the OHC in response to the BT, and that combining them would allow us extend the probe frequency range of our measurements. However, we found that suppressibility declines at different rates for the two methods as probe frequency increased. Whereas the ~ 9 dB/octave slope obtained with DPOAE suppression agrees with the spatial gradient of BM displacement as reported by Allen and Sen (2000), and confirmed by our simulation, other factors need to be considered to explain the ~ 2 dB/octave slope obtained with loudness suppression.

Allen and Sen (2000) list several animal studies, where levels of low-frequency tones required to suppress high characteristic-frequency (CF) auditory nerve responses to CF tones are almost independent of the fiber’s CF, implying a shallow slope (Abbas and Sachs 1976; Schmiedt, 1982; Fahey and Allen 1985; Delgutte 1990). A shallow slope was also noted for the psychoacoustically measured “upwards spread of masking” (Wegel and Lane 1924). Allen and Sen (2000) proposed that a spatial gradient in OHC stereocilia stiffness could create a shear motion between reticular lamina and tectorial membrane with a smaller spatial gradient than that of the BM. However, this would also imply a shallower slope for DPOAE iso-suppression, contrary to what we found in the present study. Our results are generally inconsistent with “2nd filter” theories involving the tectorial membrane, which might have had the potential to explain the shallow slope of low-side neural suppression (e.g., Allen and Fahey 1993).

More recently, Lichtenhan (2012) measured the effect of 50-Hz BM biasing on compound action potentials (CAPs) elicited by short tone pips. It is well accepted that the CAP is dominantly generated at the characteristic place. The suppressor level required to suppress the CAP in cat ears by 50 % increased by only 3–5 dB/octave as the tone-pip frequency increased, and for guinea pig ears the increase was even smaller. In addition to CAP, Lichtenhan (2012) also measured suppression of stimulus-frequency otoacoustic emissions (SFOAEs). The dependence of the suppressor levels on probe frequency was similar to that required for CAP suppression, in contrast to our DPOAE suppression data. This suggests that mechanisms underlying low-side suppression might differ between DPOAE and SFOAE (and possibly other reflection-source OAE).

**Fig. 5:**
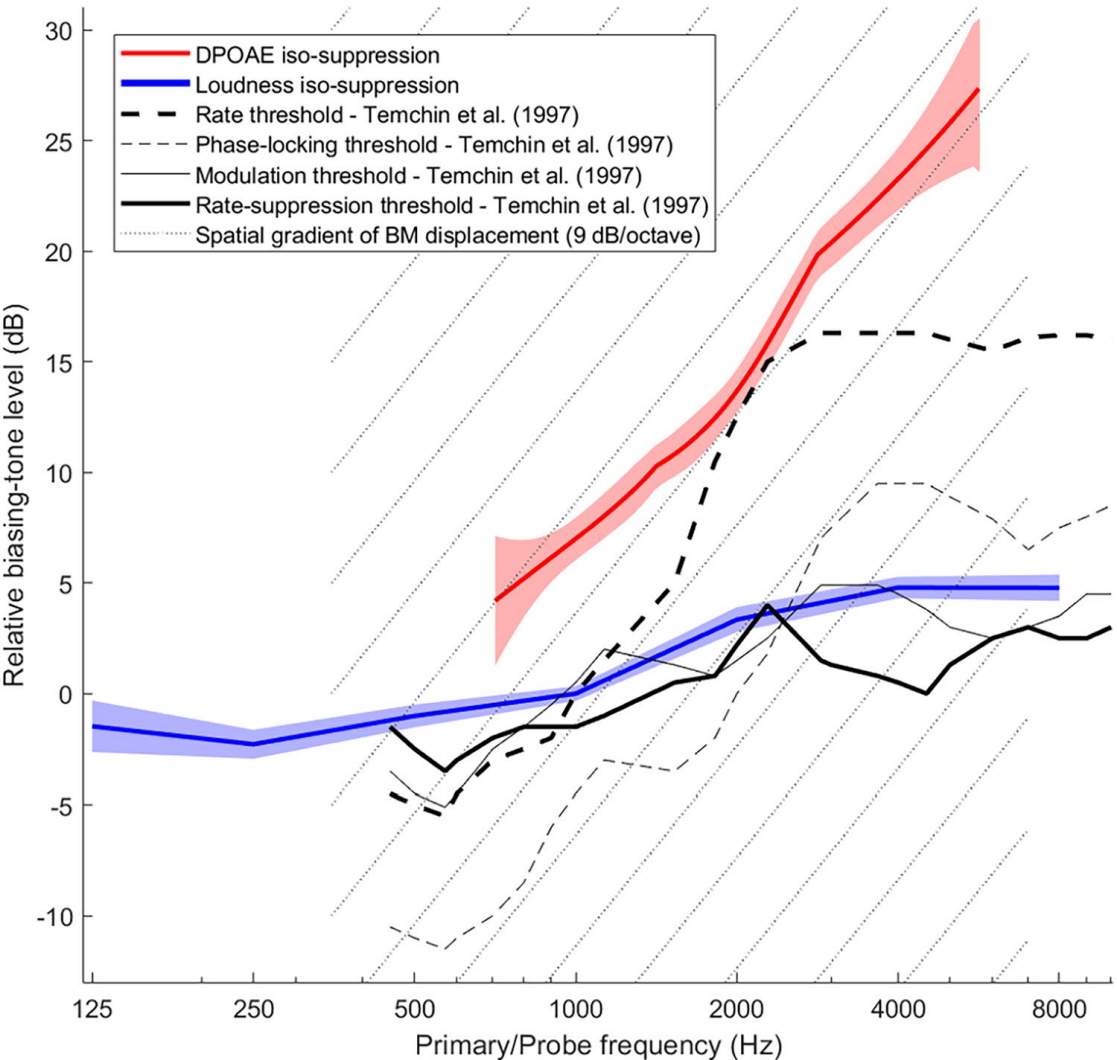
Relative BT threshold levels for DPOAE suppression (red) and loudness-suppression (blue) combined over all subjects (main and control experiments) by cumulative-slope analysis (see Methods). Shaded regions represent standard error of the means for the local slopes. The loudness iso-suppression curve was given an arbitrary level of 0 dB at 1000 Hz. The DPOAE iso-suppression curve was set higher by 7 dB at 1000 Hz, corresponding to the difference in BT levels required for DPOAE and loudness suppression at this frequency. (This difference was averaged across all 15-Hz and 30/31-Hz data from the main experiments only). For comparison, chinchilla auditory-nerve data from Fig. 11 of Temchin et al. (1997) are also shown at an arbitrary level scale as function of fiber CF, while keeping their original relative levels. Solid lines represent thresholds for suppression of CF tones. Their slopes are very similar to our loudness iso-suppression curve. Dashed lines represent thresholds to the suppressor tones alone, which had frequencies between 50 and 400 Hz. Below 2 kHz, their slopes rather match that of our DPOAE iso-suppression curve. For convenience, the grid of dotted lines indicates the slope of 9 dB/octave expected for the longitudinal gradient of BM displacement in response to the suppressor tones

Figure 5 summarizes all our DPOAE (red) and loudness (blue) iso-suppression data and compares them with data by Temchin and colleagues (1997) recorded from high-spontaneous rate fibers of the chinchilla auditory nerve (black and gray lines). Because individual subjects were sampled with different probe frequencies and ranges, we summarized the data of all individuals of both main and control experiments by deriving the shape of the iso-suppression curves via local slopes (see “Derivation of the Cumulative Average-Slope Curves”). The DPOAE data are limited to above 700 Hz, whereas loudness-suppression data extend down to 125 Hz, which was our initial motivation for performing both measurements.

Suppressor levels required to modulate or suppress an auditory nerve fiber’s response to a CF tone are shown in thin gray and bold black lines, respectively. Thresholds are roughly consistent between the two criteria, as in our data (Fig. 4). Our loudness iso-suppression curve follows these closely. The dashed lines show response thresholds to the low-frequency suppressor tones (frequencies ranging 50–400 Hz) alone as a function of fiber CF. Their threshold criteria were a modulation of the fibers’ spontaneous rate (thin), or a 20-spikes/s increase in rate (bold). Within the CF range of 0.6–2.5 kHz, the phase-locking threshold and rate-threshold curves have a slope that is reasonably close to the slope of our DPOAE iso-suppression curve, consistent with a 9 dB/octave gradient of the BM (dashed grid lines). For CFs above 3 kHz, however, these suppressor-only threshold curves flatten to a slope similar to the auditory nerve iso-suppression curves (continuous lines). Kim et al. (1979) published a spatial auditory-nerve response pattern composed from hundreds of single-fiber recordings (i.e., the neural response as a function of CF) to a 620-Hz tone of 15 dB SPL. Their pattern also flattened off completely above a CF of 3 kHz. A 180° phase jump was also seen at that CF (Kim et al. 1979). These data suggest that the high-CF fibers are not mechanically activated by the local 9-dB/octave travelling wave tail, but may be driven by the same mechanism that also underlies low-side neural suppression.

A bias in OHC stereocilia angle is the widely accepted reason for the suppression of DPOAE. The stereocilia are deflected by the shear motion between reticular lamina and tectorial membrane. Our interpretation of DPOAE suppression, as reflecting BM displacement, thus supposes that shear and BM displacement have the same spatial gradient. This does not agree with measurements obtained by phase-sensitive optical coherence interferometry, that have shown that the frequency-dependence of reticular lamina vibration below CF is apparently less steep than that of BM vibration (e.g., He et al. 2018; Dewey et al. 2019). It has been, however, suggested that these reticular lamina vibration data could be strongly impacted by longitudinal fluid motion within the organ of Corti (Cooper et al. 2018). In our view, it is not clear yet whether the published reticular lamina tuning-curve slopes are indeed representing the transversal reticular lamina motion.

Guinan (2012) postulated the existence of multiple and rather complex mechanical ways of IHC stimulation, some of which imply differential movements of OHC and IHC stereocilia. Very likely, however, the free-standing IHC stereocilia do not experience any effective bias from the extremely low-frequency BTs used in our experiments. A non-mechanical explanation for neural low-side suppression, based on extracellular potentials, seems to us therefore more plausible. Salt and colleagues (2013) demonstrated experimentally the extraordinary effectiveness of very low-frequency tones to generate large electrical potentials within the cochlear ducts, probably facilitated by the in-phase motion of the BM and the accordingly synchronized OHC-currents along the entire cochlea. They also demonstrated pharmacologically that the most apically located OHCs are the main generators of these potentials, consistent with the 9-dB/octave BM gradient. It has often been suggested that these OHC-generated potentials may exceed intrinsic IHC-receptor potentials and thus influence or even trigger synaptic transmission (e.g., Sellick et al. 1982; Russell and Sellick 1983; Ruggero et al. 1986; Cheatham and Dallos 1997). The conductivity of the ionic fluid lets the potentials spread with a far shallower gradient than the mechanical gradient along the BM. Salt and colleagues (2013) showed that the electrical fields in the guinea-pig cochlea decay by only ~ 20 dB between the 3rd and 1st turn, a CF range of at least 5 octaves (i.e., < 4 dB/octave). It is therefore conceivable that the shallow slope of low-side neural suppression reflects the shallow gradient of these electrical fields. If so, loudness suppression is not a reliable measure of BM displacement, contrary to our initial assumption.

Irrespective of the underlying factors, it is remarkable that 3 of 12 subjects could detect the modulation of a 125-Hz or 250-Hz probe by a 15-Hz BT at levels for which the BT was undetectable in isolation (control experiments). Averaged over subjects, modulation threshold was just ~ 5 dB SL at these frequencies, and remained below ~ 10 dB SL across the full range of probe frequencies (Fig. 4). In other words, a 15-Hz tone of just 10 dB SL has the capacity to modulate the neural responses to higher-frequency sounds across almost the entire auditory spectrum. The main experiments, which used the more stringent criterion of full suppression (Fig. 2B), found higher dB SL thresholds for complete loudness suppression with the 31-Hz BT than with the 7- and 15-Hz BTs. Extrapolating, one could speculate that the suppression-threshold levels will exceed the sensation-threshold levels even further as BT frequency increases. This is expected from a 6 dB/octave increase in BT sensation due to the IHC's sensitivity to velocity, in contrast to displacement-sensitive OHCs, which are the main-generators of the extracellular potentials that we believe underlie low-side neural suppression. Thus, infrasound is special in that it can be a low-side suppressor that is hardly perceptible by itself.

The modulatory effect on higher-frequency sounds of a low-frequency sound that is itself hardly perceptible seems relevant for debates centered on the often-reported annoyance caused by low-frequency noise pollution. It could account for the puzzling discrepancy between subjective reports of annoyance, and objective acoustic measurements that show levels of low-frequency sound near or even below detection threshold. If so, modulation of sounds in the conventional range of hearing might underlie some of the complaints about environmental noise with strong low-frequency content. Given its importance, this interpretation needs to be confirmed by dedicated experiments.

## Data Availability

All data generated or analyzed during this study are included in the manuscript.
